# The Association between Renal Hyperfiltration and the Sources of Habitual Protein Intake and Dietary Acid Load in a General Population with Preserved Renal Function: The KoGES Study

**DOI:** 10.1371/journal.pone.0166495

**Published:** 2016-11-15

**Authors:** Rina So, Sihan Song, Jung Eun Lee, Hyung-Jin Yoon

**Affiliations:** 1 Department of Biomedical Engineering, Seoul National University College of Medicine, Seoul, Republic of Korea; 2 Department of Food and Nutrition, Sookmyung Women’s University, Seoul, Republic of Korea; 3 Department of Food and Nutrition, Seoul National University College of Human Ecology, Seoul, Republic of Korea; The University of Tokyo, JAPAN

## Abstract

Although the differential response of the kidney to the acute load of various sources of dietary protein in subjects with normal renal function is well known, the influence of habitual dietary protein intake and dietary acid load on renal function has not been tested well. The association between renal hyperfiltration (RHF), the earlier and possibly reversible stage of chronic kidney disease, and the sources of habitual dietary protein and dietary acid load was analyzed with the baseline data of 123,169 middle-aged healthy Koreans of a large prospective cohort study, who had a baseline estimated glomerular filtration rate (eGFR) >60 mL/min/m^2^ and no known history of diabetes and/or hypertension. eGFR was calculated with the Chronic Kidney Disease Epidemiology Collaboration equation using serum creatinine and RHF was defined as eGFR >95^th^ percentile after adjustment for age, sex, height, and body weight. Dietary acid load was calculated with estimated net endogenous acid production (eNEAP). Although the level of habitual intake of animal protein was positively and vegetable protein was negatively associated with RHF, this association was significant only in women and younger participants (younger than sex-specific median age). The odds for RHF increased as the percentile rank of eNEAP increased until about the 50^th^ percentile and then leveled off. The positive association between eNEAP and RHF was significant in both sexes and age groups. Dietary acid load was associated with RHF regardless of sex and age and rather than the amount of the total or the individual sources of habitual dietary protein, may be a better target for the dietary intervention of chronic kidney disease.

## Introduction

The Western-style diet, characterized by higher consumption of animal products and lower consumption of vegetables and fruits, has been associated with many clinical conditions, including chronic kidney disease (CKD) [1–3]. CKD is becoming one of the most important global health issues because of its association with cardiovascular diseases and its rapidly increasing incidence and prevalence worldwide, mainly due to an ageing population and an epidemic of obesity and diabetes. Although the Western-style diet is known as one of the main risk factors for the progression of CKD and is recommended to be avoided in the patients with CKD [2, 4], its role(s) in the development of CKD is not clear yet.

Based on the facts that acute protein loading cause an increase and dietary protein restriction caused a decrease in glomerular filtration rate (GFR) of healthy subjects [5, 6], dietary protein is believed to be one of the main modulators of GFR in physiological condition and to be associated with the increased risk of CKD development and progression through renal hyperfiltration (RHF) [7]. However, the direct evidence in human is not consistent, especially in the setting of habitual protein intake [8, 3]. RHF, one of the mechanisms of CKD progression and possibly an earlier, reversible stage of CKD, has been suggested as an all-cause mortality marker in a healthy general population [9]. The association between RHF and habitual dietary protein intake has not been tested.

Previous single-meal studies and short-term clinical trials found that vegetable protein caused a less or no increase in GFR in persons with normal renal function, compared to animal protein [10–12]. Because it has been suggested that not only the amount but also the duration of exposure to dietary protein might influence the renal response to dietary protein [13], the observations in single meal studies or short-term clinical trials must be confirmed by studies on the habitual protein intake. There are only a few reports on renal function concerning the sources of habitual dietary protein intake, and the results are not consistent [14, 15]. Although animal protein is the main source of dietary acid load [1], of which markers have been associated with RHF [16], the association between dietary acid load and RHF has not been studied yet.

To study the association between RHF and the sources of habitual dietary protein and dietary acid load, the baseline data of healthy adult participants of a large prospective cohort study in Korea (Korean Genome and Epidemiology Study, KoGES study) were analyzed.

## Subjects and Methods

### Study population

The Korean Genome and Epidemiology Study (KoGES), recruited 173,357 participants who visited one of 39 hospitals in Korea between November 2004 and December 2013 for a health check-up, is a prospective cohort study of which the primary goal was to elucidate the interaction between lifestyle factors and genetic risk factors in the development of noncommunicable diseases, including CKD [17]. The participants, who were aged between 40 and 70 years, provided written informed consent to participate in this study. The data on alcohol intake, smoking status, past medical history, socioeconomic status, physical activity, and diet were collected through face-to-face interview using a structured questionnaire. The interviewers at each participating center completed regular training courses to ensure a standardized interview process throughout the study. The data of 15,633 participants were excluded because of incomplete data including those of 3,066 participants who submitted no information on the food frequency questionnaire and 1,818 participants with implausible energy intake above the log-transformed mean±3 standard deviation, known histories of malignant tumors or estimated GFR calculated by the Chronic Kidney Disease Epidemiology equation (CKD-EPI; eGFR) <60 mL/min/1.73 m^2^. After further exclusion of 34,555 participants with a known history of hypertension and/or diabetes, we analyzed the health screening data of 39,792 men and 83,377 women.

Data in this study were from the Korean Genome and Epidemiology Study (KoGES; 4851–302), National Research Institute of Health, Centers for Disease Control and Prevention, the Ministry for Health and Welfare, Republic of Korea. The institutional review board of Seoul National University Hospital exempted the approval process because the data were publically available.

### Anthropometric and laboratory measurements

Blood pressure was measured on the left arm with mercury sphygmomanometers after 5 minutes of rest. Two measurements taken at least 1 minute apart were averaged. Body mass index (BMI) was calculated by dividing the body weight by height squared (kg/m^2^).

Blood and urine samples were taken after at least 8 hours of fasting and analyzed by the central laboratory. The serum creatinine level was measured by the Jaffé method using Cobas 8000 C702-1 (Roche Diagnostics, Basel, Switzerland). The coefficient of variance was 0.82–5.46%. The serum creatinine concentration was standardized to isotope dilution mass spectrometry as previously proposed [18]. The eGFR was calculated with the CKD-EPI [19] and RHF was defined as eGFR >95^th^ percentile in the distribution of residuals from a multiple linear regression where logarithm-transformed eGFR, respectively, was the dependent variable, and sex, height, weight, and logarithm-transformed age were the independent variables [20]. Albuminuria was defined as 1+ or higher on the dipstick urine test for proteinuria performed on fasting spot urine. Smoking history was classified as current smokers, ex-smokers, and non-smokers. Regarding regular alcohol consumption, the subjects were categorized into two groups; non-drinkers and current drinkers. Exercise status was defined as regular exercise or no regular exercise.

### Assessment of dietary intake

The dietary intake of the subjects during the year prior to enrollment was assessed using a food-frequency questionnaire comprising 103 food items that was developed to assess the usual dietary intake of Korean adults. Details of this questionnaire, including its relative validity, have been published [21]. In brief, participants were asked about how often, on average, they had consumed the listed food items during the previous year. The frequency of servings was classified into nine categories ranging from “never or seldom” to “three times or more a day,” and portion sizes were categorized into small, medium, or large. Daily nutrient intakes were estimated by multiplying the nutrient content by the serving per day of each food, and summing over all foods. Dietary acid load was estimated using the equation for net endogenous acid production (eNEAP), described by Frassetto et al: eNEAP (mEq/day) = [54.5 x protein intake (g/day) ÷ potassium intake (mEq/day)]– 10.2 [22]. The level of daily nutrient intake was divided into quartiles according to the gender.

### Statistical analysis

All statistical analysis was conducted using SAS version 9.3 (SAS Institute Inc., Cary, NC, USA) and R software 3.3.0 (http://www.R-project.org). Continuous variables were compared with t-test and discrete variables with chi-squared test. To examine the association between the prevalence of RHF and sex-specific quartiles of energy-adjusted nutrient and eNEAP, odds ratios (ORs) and 95% confidence intervals (CIs) were obtained using multivariate logistic regression with adjustment for possible confounding variables, such as regular exercise (yes or no), smoking (non-smoker, ex-smoker, or current smoker), alcohol consumption (quartiles), systolic blood pressure (continuous), serum fasting glucose (continuous), serum triglyceride (continuous), serum high density lipoprotein-cholesterol (HDP-chol; continuous), albuminuria (negative or positive, and total energy intake (kcal per day, quartiles). Intakes of nutrients were energy-adjusted by using the residuals from a regression model with caloric intake as the independent variable and the nutrient intake as the dependent variable [23]. We also examined the effect of substituting energy-adjusted nutrients from animal sources for the same amount of vegetable sources. All tests were two-sided and P values lower than 0.05 were considered statistically significant.

## Results

The general characteristics of the participants are showed in Table 1. The mean age of men without RHF and those with RHF was 51.8±0.0 years and 52.2±0.2 years, respectively and that of women without RHF and those with RHF was 50.9±0.0 years and 52.3±0.1 years, respectively. Mean eNEAP of men without and with RHF was 52.8±0.1 mEq/day and 53.7±0.4 mEq/day, respectively and that of women without and with RHF was 47.2±0.1 mEq/day and 48.1±0.2 mEq/day, respectively. The proportion of participants older than sex-specific median age, those with BMI higher than 25 kg/m^2^, and those who did not exercise regularly was higher in participants with RHF than in those without RHF, in both men and women. The proportion of current smokers and habitual drinkers was higher in men with RHF than in those without RHF. Systolic blood pressure, and fasting serum glucose and triglyceride was higher in participants with RHF than in those without RHF, in both men and women. The proportion of albuminuria was not different both in those without and with RHF (Table 1).

**Table 1:** General characteristics of the participants^1^.

		Men				Women			
Characteristic		Without RHF^2^	With RHF^2^	Total	P Value^3^	Without RHF^2^	With RHF^2^	Total	P Value^3^
		(n = 37,802)	(n = 1,990)	(n = 39,792)		(n = 79,208)	(n = 4,169)	(n = 83,377)	
Age		51.8±0.0	52.2±0.2	51.9±0.0	0.0725	50.9±0.0	52.3±0.1	51.0±0.0	< .0001
The older^4^, %		48.8	51.9	49.0	0.0079	48.6	59.9	49.2	< .0001
Body mass index		24.1±0.0	24.8±0.1	24.2±0.0	< .0001	23.3±0.0	23.6±0.0	23.3±0.0	< .0001
Overweight^5^, %		35.9	46.0	36.4	< .0001	24.7	26.6	24.7	0.0054
Smoking, %					< .0001				0.0473
	Nonsmoker	27.8	21.7	27.5		96.3	95.6	96.3	
	Ex-smoker	37.9	36.0	37.8		1.3	1.5	1.3	
	Current smoker	34.3	42.3	34.7		2.4	2.9	2.4	
Regular exercise, %		54.9	47.3	54.6	< .0001	50.1	45.6	49.9	< .0001
Current drinker, %		73.8	78.2	74.0	< .0001	33.6	34.1	33.6	0.5175
Systolic blood pressure (mmHg)		123.8±0.1	126.1±0.3	123.9±0.1	< .0001	118.3±0.1	119.9±0.2	118.4±0.1	< .0001
Diastolic blood pressure (mmHg)		77.9±0.0	78.6±0.2	78.0±0.0	0.0060	73.6±0.0	73.7±0.1	73.6±0.0	0.7402
Fasting serum glucose		94.7±0.1	98.9±0.5	94.9±0.1	< .0001	90.1±0.0	91.4±0.2	90.2±0.0	< .0001
Serum triglyceride (mg/dL)		147.7±0.5	161.9±3.1	148.4±0.5	< .0001	107.2±0.2	111.3±1.2	107.5±0.2	0.0013
Serum HDL-Chol^6^ (mg/dL)		50.2±0.1	50.6±0.3	50.2±0.1	0.1721	57.2±0.0	56.1±0.2	57.1±0.0	< .0001
Albuminuria^7^, %		1.9	2.4	2.0	0.1710	1.9	2.0	1.9	0.6508
eGFR^8^ (mL/min/1.73 m^2^)		93.2±0.1	109.7±0.1	94.1±0.1	< .0001	96.7±0.0	110.9±0.1	97.4±0.0	< .0001
eNEAP^9^ (mEq/day)		52.8±0.1	53.7±0.4	52.9±0.1	0.0156	47.2±0.1	48.1±0.2	47.2±0.1	< .0001
Total energy intake (kcal/day)		1868.2±2.7	1882.7±12.2	1869.0±2.6	0.2269	1721.0±1.9	1715.1±8.3	1720.7±1.8	0.4819

1. Mean±standard error for continuous variables and % for discrete variables

2. Renal hyperfiltration (see Subjects and Methods for details)

3. t-test for continuous data and chi-square test for discrete variables

4. Older than the sex-specific median age (50 years in men, 51 years in women)

5. Body mass index >25 kg/m^2^

6. Serum high density lipoprotein cholesterol

7. Proteinuria by dipstick analysis 1+ or higher

8. Glomerular filtration rate estimated by the Chronic Kidney Disease Epidemiology Collaboration creatinine equation (see Subjects and Methods for details)

9. Estimated net endogenous acid production (see Subjects and Methods for details)

**Fig 1:**
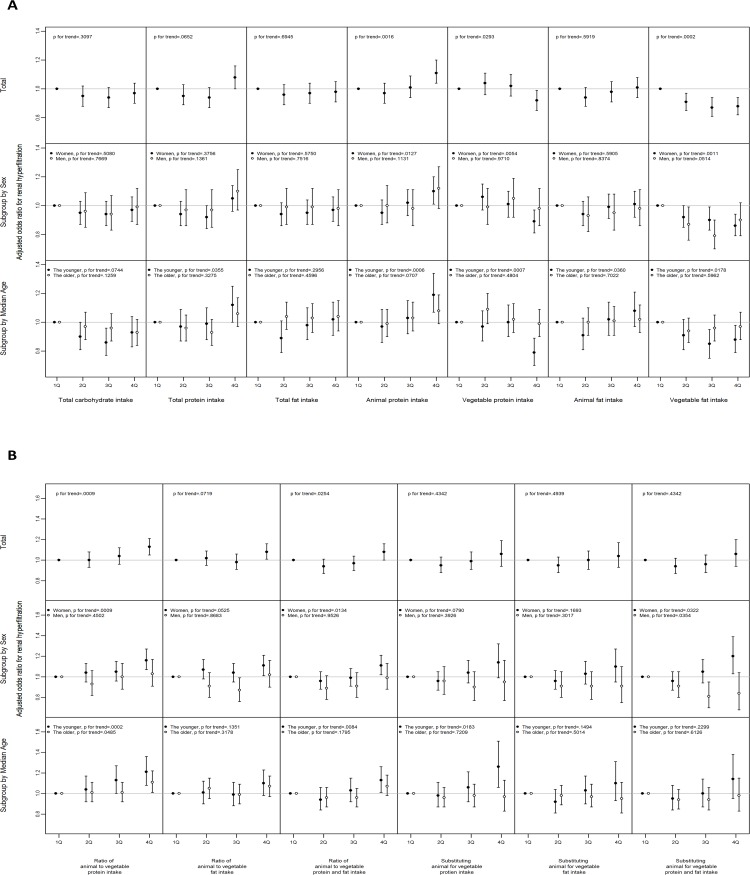
The association between renal hyperfiltration and the components of habitual dietary intake. (A) The level of total intake of carbohydrate, protein and its sources, and fat and its sources. (B) The ratio of animal to vegetable sources of protein and fat and the substitution model. All logistic regression analyses were adjusted for systolic blood pressure, alcohol intake, smoking status, regular exercise, fasting serum glucose, serum triglyceride, serum high density lipoprotein cholesterol, and albuminuria. The median age of men was 51 years and that of women was 50 years. Error bars mean 95% confidence interval. See Subjects and Methods for the definition of renal hyperfiltration.

Although the level of habitual intake of total protein was not significantly associated with RHF both in either men (OR for RHF 1.10, 95% CI 0.97~1.25 in the highest quartile compared to the lowest quartile; p for trend>0.05) or women (1.05, 0.96~1.14; p for trend>0.05), animal protein intake level was positively and vegetable protein intake was inversely associated with the odds of RHF in women (1.10, 1.01~1.20; p for trend = 0.013 for animal protein; 0.89, 0.81~0.97, p for trend = 0.005 for vegetable protein) and participants younger than sex-specific median age (1.19, 1.07~1.34, p for trend = 0.001 for animal protein; 0.79, 0.70~0.89, p for trend = 0.001 for vegetable protein; Fig 1A). The ratio of animal to vegetable protein intake was positively associated with the odds of RHF only in women (1.16, 1.07~1.27, p for trend = 0.001). The ratio was positively associated with RHF irrespective of the age (1.21, 1.08~1.35, p for trend<0.001, in the younger participants; 1.11, 1.01~1.22, p for trend = 0.049 in the older participants; Fig 1B). The level of substitution of the energy from vegetable protein with the energy from animal protein was not associated with the odds of RHF both in either men or women, but positively associated in the younger participants (1.26, 1.06~1.51, p for trend = 0.018; Fig 1B). The source of habitual dietary protein, the ratio of animal to vegetable protein intake, and the level of substitution of the vegetable protein with the animal protein were not associated with the odds of RHF in men and the older participants (Fig 1B).

**Fig 2:**
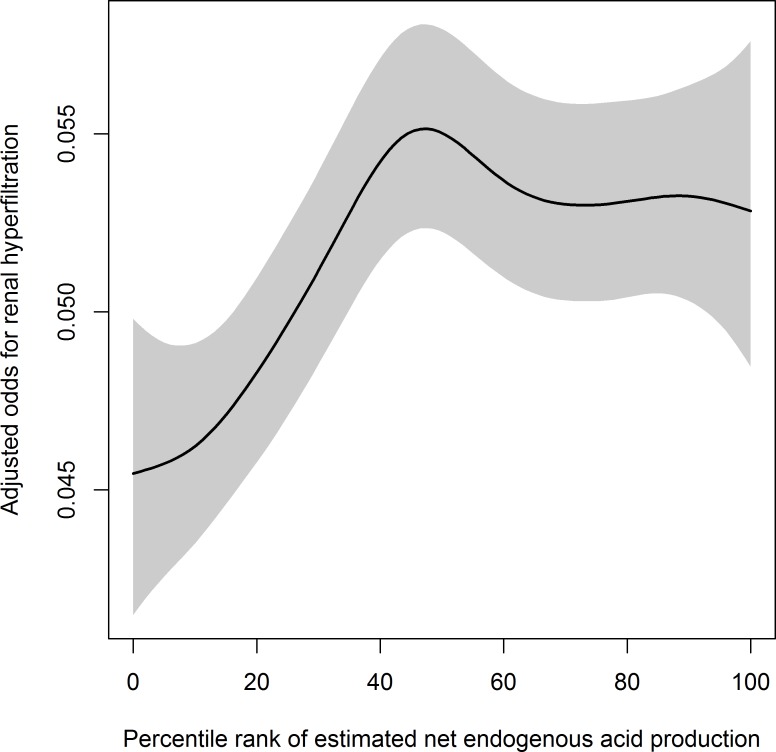
The association between renal hyperfiltration and the percentile rank of estimated net endogenous acid production (NEAP). Logistic regression analysis was adjusted for systolic blood pressure, alcohol intake, smoking status, regular exercise, fasting serum glucose, serum triglyceride, serum high density lipoprotein cholesterol, and albuminuria. Penalized splines were used for smoothing and the degree of freedom of splines was selected by a generalized cross validation method. Shaded area means 95% confidence interval. See Subjects and Methods for the definition of renal hyperfiltration and NEAP.

**Fig 3:**
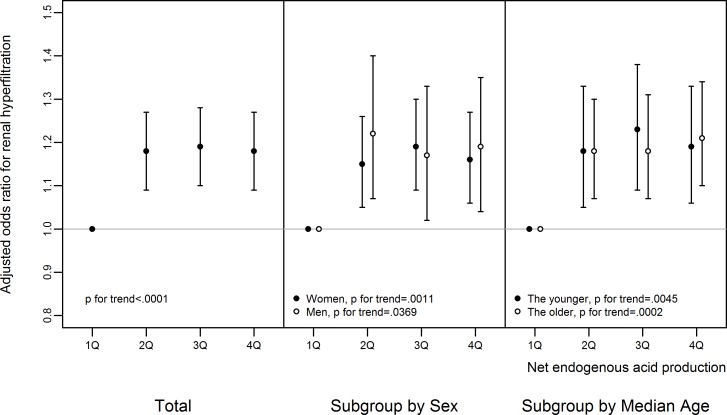
The association between renal hyperfiltration and estimated net endogenous acid production. All the logistic regression analyses were adjusted for systolic blood pressure, alcohol intake, smoking status, regular exercise, fasting serum glucose, serum high density lipoprotein cholesterol, and albuminuria. The median age for men was 51 years and that of women was 50 years. Error bars mean 95% confidence interval. See Subjects and Methods for the definition of renal hyperfiltration and estimated net endogenous acid production.

The dietary acid load was estimated with eNEAP and the percentile rank of eNEAP was associated with the odds of RHF. The odds of RHF was increased with the increase of eNEAP percentile rank up to about the 50^th^ percentile and then leveled off (Fig 2). Compared to the association between the source of habitual dietary protein intake and RHF, which was significantly associated only in women and the younger participants, the level of eNEAP was positively associated with RHF irrespective of sex and age (1.19, 1.04~1.35, p for trend = 0.037 for men; 1.16, 1.06~1.27, p for trend = 0.001 for women; 1.19, 1.06~1.33, p for trend = 0.005 for the younger; 1.21, 1.10~1.34, p for trend<0.001 for the older; Fig 3). The pattern of association between eNEAP and RHF was similar according to BMI (25 kg/m^2^ or lower and higher than 25 kg/m^2^) and the smoking status (non-smokers, ex-smokers, and current smokers) (data not shown). eNEAP was positively correlated with animal protein intake, negatively with total protein and vegetable protein intake. The correlation coefficient between eNEAP and animal protein intake was 0.01 (95% CI, 0.01~0.02), total protein intake, -0.11 (95% CI, -0.11~-0.10), vegetable protein intake, -0.32 (95% CI, -0.33~-0.32), and ratio of animal to vegetable protein intake, 0.09 (95% CI, 0.09~0.10)(data not shown). No significant difference in this correlation was observed between men and women.

## Discussion

This study, on a large cohort of healthy middle aged adults with preserved renal function and no history of hypertension and/or diabetes found that the level of habitual intake of animal protein was associated with higher odds of RHF in women and the participants younger than the sex-specific median age and that a higher dietary acid load was associated with higher odds of RHF irrespective of sex and age. Although the main source of dietary acid load is protein from animal sources, dietary acid load may be a better marker indicating RHF associated with the habitual dietary pattern than the total amount or sources of habitual dietary protein.

RHF, an early reversible stage of chronic kidney disease, has been suggested as an all-cause mortality marker [9], and associated not only with many renal conditions but also with various lifestyle factors such as smoking [24], lack of physical activity [25], and lower cardiopulmonary fitness [26]. RHF is known as one of the mechanisms in the progression of chronic renal disorders to end-stage renal failure and higher dietary protein intake is believed to be one of the main causes of RHF [27], of which evidence in human is scarce though. The lack of the consensus on the definition of RHF may partly be the cause of the absence of evidence in human [28, 29]. RHF, despite of the lack of universally accepted definition of RHF, was defined in this study as eGFR with residuals >95^th^ percentile, because RHF defined by the same cutoff value was associated with lower serum bicarbonate levels and higher all-cause mortality in healthy Korean adults [9, 16]. The prevalence of albuminuria was not different between the participants with and without RHF in this study. The association between RHF and albuminuria in subjects other than those with diabetes is not clear. RHF in diabetes is believed to be followed by albuminuria and the possible pathophysiological independency of RHF upon albuminuria in diabetes has been suggested [30, 31]. In pediatric patients with nephron-urological disorders, microalbuminuria was not associated with RHF [32].

Most of previous studies on the association between renal function and dietary protein intake observed the change of GFR in the response to test meals of relatively short duration [5, 6], and the association between renal function and habitual intake of dietary protein has not been studied well. Brändle et al. reported a positive association between urinary nitrogen excretion rate and endogenous creatinine clearance in 88 healthy volunteers with normal renal function, who were on their diet for at least 4 months [33]. An absence of the association between the intake level of total protein and eGFR in 2419 postmenopausal women has been reported [34], and our observation was consistent to this observation. Several single meal studies reported an association of an increase in GFR with the animal source of protein, but not with the vegetable source, such as soy protein [10, 12] or an alleviation of RHF after a soy protein-rich diet [11]. The differential response of kidney to the sources of habitual protein intake observed in our study was consistent with these single meal studies. The reports on the association between the sources of habitual dietary protein and renal function are not consistent. Although a cross-sectional study reported that measured GFR of vegans was lower than that of omnivores and that of lactovegetarians was in between [15], a cross-sectional study from Taiwan reported that eGFR of vegetarians was not significantly different from that of omnivores [14]. Our study observed a significant association between RHF and the sources of habitual dietary protein only in women and relatively younger participants, and the inconsistency of previous studies might be resulting from the difference in sex and/or age distribution among the participants between the studies.

Although there are several explanations of the differential association between RHF and the sources of dietary protein, including differential hormonal responses, such as prostaglandin and glucagon [12], the exact mechanism is yet to be settled. Dietary acid load has been associated with many clinical conditions, including a higher risk of incident CKD in general population [35, 36] and progression of CKD to end-stage renal failure [37]. Although dietary animal protein is the main source of dietary acid load, and the marker of dietary acid load such as lower serum bicarbonate has been associated with RHF in subjects with preserved renal function has been reported [16], the association between dietary acid load and renal function or RHF in general population without CKD has not been studied. For the first time, our observation provides evidence that dietary acid load may be another possible explanation of the association between RHF and the sources of dietary protein.

With subgroup analysis, the difference in the odds for RHF according to the sources of dietary protein was observed only in women and the participants younger than the sex-specific median age. Subgroup analyses according to BMI and smoking status did not exhibit any difference. Previous studies of the association between the sources of dietary protein and GFR did not analyze the possible gender and age difference and our observations need to be confirmed. Because the association between RHF and dietary acid load was irrespective of sex and age, the difference in the overall dietary composition other than dietary protein between sex and age groups may explain the absence of the association between RHF and the sources of habitual dietary protein intake in men and the older participants.

This study has several limitations. First, GFR was estimated, not measured. Measurement of GFR in a very large prospective cohort was not practically possible. Second, the measurement of biomarkers representing dietary acid load was not provided. Although the estimation equation used in this study has been widely adopted in many studies including those performed on populations other than Western countries, it has been validated in Western population, not in Asians [38]. Third, the causal relationship between the dietary acid load and RHF could not be tested due to the observational nature of this study. Fourth, the observation in this study was performed on a single ethnic group and the generalization of it should be cautious.

Despite of these limitations, an association between RHF and the sources of habitual dietary protein in women and young participants and an association between RHF and dietary acid load regardless of sex and age has been observed in this study on a very large prospective cohort with preserved renal function and with no known history of diabetes and/or hypertension. Regarding the clinical implications of RHF as a mortality risk factor and as an early and reversible stage of CKD, the observations of this study need to be confirmed through intervention studies such as dietary modification or alkali supplement. Dietary acid load, rather than the amount of the total or the individual sources of dietary protein, may be a better target for dietary intervention and prevention of CKD.
